# The first report on brain sagging dementia caused by a cranial leak: A case report

**DOI:** 10.3389/fneur.2022.1006060

**Published:** 2022-09-29

**Authors:** Aslan Lashkarivand, Per Kristian Eide

**Affiliations:** ^1^Department of Neurosurgery, Oslo University Hospital – Rikshospitalet, Oslo, Norway; ^2^Institute of Clinical Medicine, Faculty of Medicine, University of Oslo, Oslo, Norway

**Keywords:** cranial, CSF leak, brain, sagging, dementia, case report

## Abstract

**Objective:**

Brain Sagging Dementia (BSD) is an increasingly recognized syndrome for which diagnostic criteria recently were proposed. There have been no reports on BSD caused by a cranial leak. Here we present the first report on a patient with BSD caused by a cranial leak.

**Case description:**

A 60-year old male patient was admitted with a 2-year history of orthostatic headache and gradually progressive cognitive and behavioral changes. Traditional treatments for spontaneous intracranial hypotension, including repeated epidural blood patches, failed. Brain imaging showed severe brain sagging, and intracranial pressure monitoring demonstrated intracranial hypotension. No leakage site was found. His past medical history revealed an accident where a ski pole struck his head at age ten. Due to progressive clinical decline, surgery was pursued. A cranial defect with an accompanying cerebrospinal fluid leak site representing the trauma from his childhood was found and repaired. He also was in need of a ventriculoperitoneal shunt. Following surgery, he improved and recovered completely.

**Discussion:**

This case report illustrates that a cranial leak may cause BSD, even with a “lucid interval” between trauma and symptom debut spanning many years. Moreover, this report validates well the recently proposed BSD diagnostic criteria.

## Introduction

Brain sagging dementia (BSD) is a rare syndrome that results in behavioral and cognitive changes mimicking behavioral variant frontotemporal dementia (bvFTD) (1, 2). Spontaneous intracranial hypotension (SIH) caused by cerebrospinal fluid (CSF) leakage is thought to be the cause, however, no cranial leaks have been reported (3, 4). We present the first report on a patient fulfilling the recently proposed BSD diagnostic criteria (3), caused by a cranial CSF leakage.

## Methods

The review was reported according to PRISMA guidelines and registered with the PROSPERO database CRD42020150709 (see Supplementary Table S1 and Supplementary Figure S1). The search was updated in May 2022 (see Supplementary material). The case report was reported according to CARE guidelines.

## Case description

**Figure 1:**
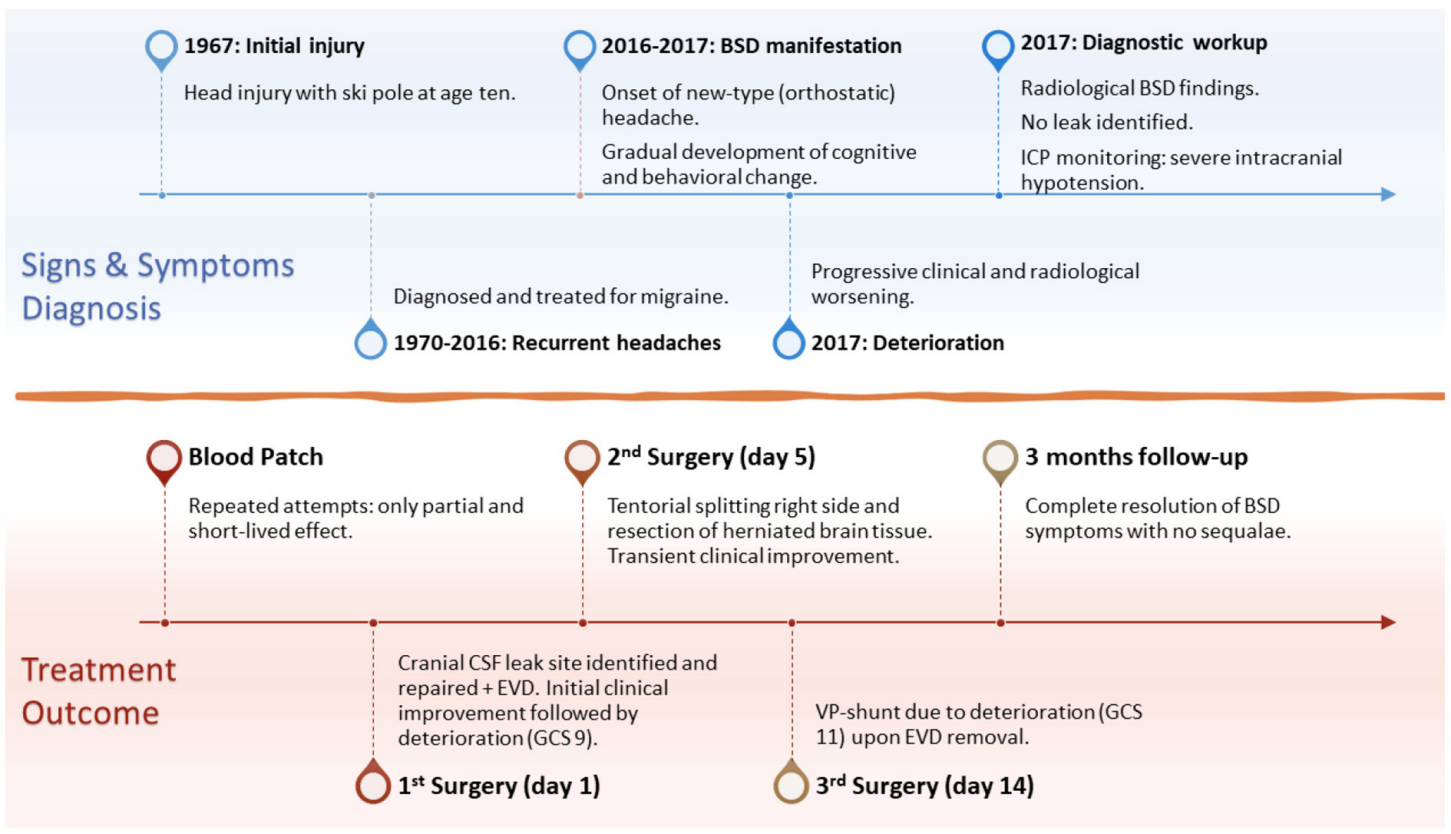
Timeline of the disease development and progression, signs and symptoms, diagnostic workup and findings, surgical treatment, and 3-months follow-up. BSD, brain sagging dementia; ICP, intracranial pressure; EVD, external ventricular drain; GCS, Glasgow Coma Scale; VP-shunt, ventriculoperitoneal shunt.

A 60-year-old Caucasian man was referred to our department in 2017 for a second opinion due to failed response to traditional SIH treatment. About 1 year earlier, he was admitted to the neurological department due to orthostatic headache and subtle cognitive changes recognized by his family members. Craniospinal magnetic resonance imaging (MRI) showed signs of SIH; however, no CSF leak was evident. Lumbar puncture showed a low opening pressure (near zero cmH_2_O). Repeated attempts with lumbar epidural blood patch during the next few months had only partial and short-lived effects. Meanwhile, his condition gradually progressed. His past medical history was uneventful, except for a head injury at age ten when a ski pole struck his left temporal head region. He was discharged from hospital after 2 days with no great concern and no signs of skull fracture at X-ray. As an adolescent, he developed tension headaches (worst in supine position) that worsened during early adult life. In his 30 s, he was once hospitalized, diagnosed with migraine, and medicated for this until 2016. He then suffered a “new type of headache” that was worse in standing position (Figure 1). Thereafter, his condition progressed rapidly, manifesting a fulminant BSD (Table 1), with severe cognitive decline confirmed by neuropsychological assessment. He failed to perform at his job as a company director, and was on full sick leave.

**Table 1:** Diagnostic criteria for brain sagging dementia (BSD) (3).

**Absolute clinical and imaging criteria**	**Present**	**Absent**
Signs and symptoms of bvFTD (1, 2)^*^	✓	
Absence of bvFTD imaging findings; frontotemporal atrophy^†^	✓	
Imaging findings of brain sagging	✓	
Insidious onset, and slowly progressing (>3/4 weeks)	✓	
No history of recent trauma or lumbar puncture	✓	
Symptom onset before 65 years of age	✓	
Symptoms cannot be explained by altered level of consciousness alone	✓	
At least one of the supporting clinical criteria (SIH) or 3 of the additional criteria	✓	
**Supporting clinical criteria**		
Orthostatic headache	✓	
Low lumbar puncture opening pressure	✓	
Improvement of symptoms after blood patch	✓	
**Additional criteria**		
Headache	✓	
Cerebellar signs and symptoms	✓	
Hypersomnolence	✓	
Choreiform movements		×
Pachymeningeal enhancement on imaging	✓	
Subdural effusion on imaging	✓	
Evidence of cerebrospinal fluid leak on myelogram		×

(1, 2) bvFTD, behavioral variant frontotemporal dementia; SIH, spontaneous intracranial hypotension.

* Signs and symptoms must meet the diagnostic criteria of bvFTD.

† Frontotemporal atrophy must be ruled out, while findings on PET and SPECT will not alter the diagnosis.

The International Classification of Headache Disorders, 3rd edition.

**Figure 2:**
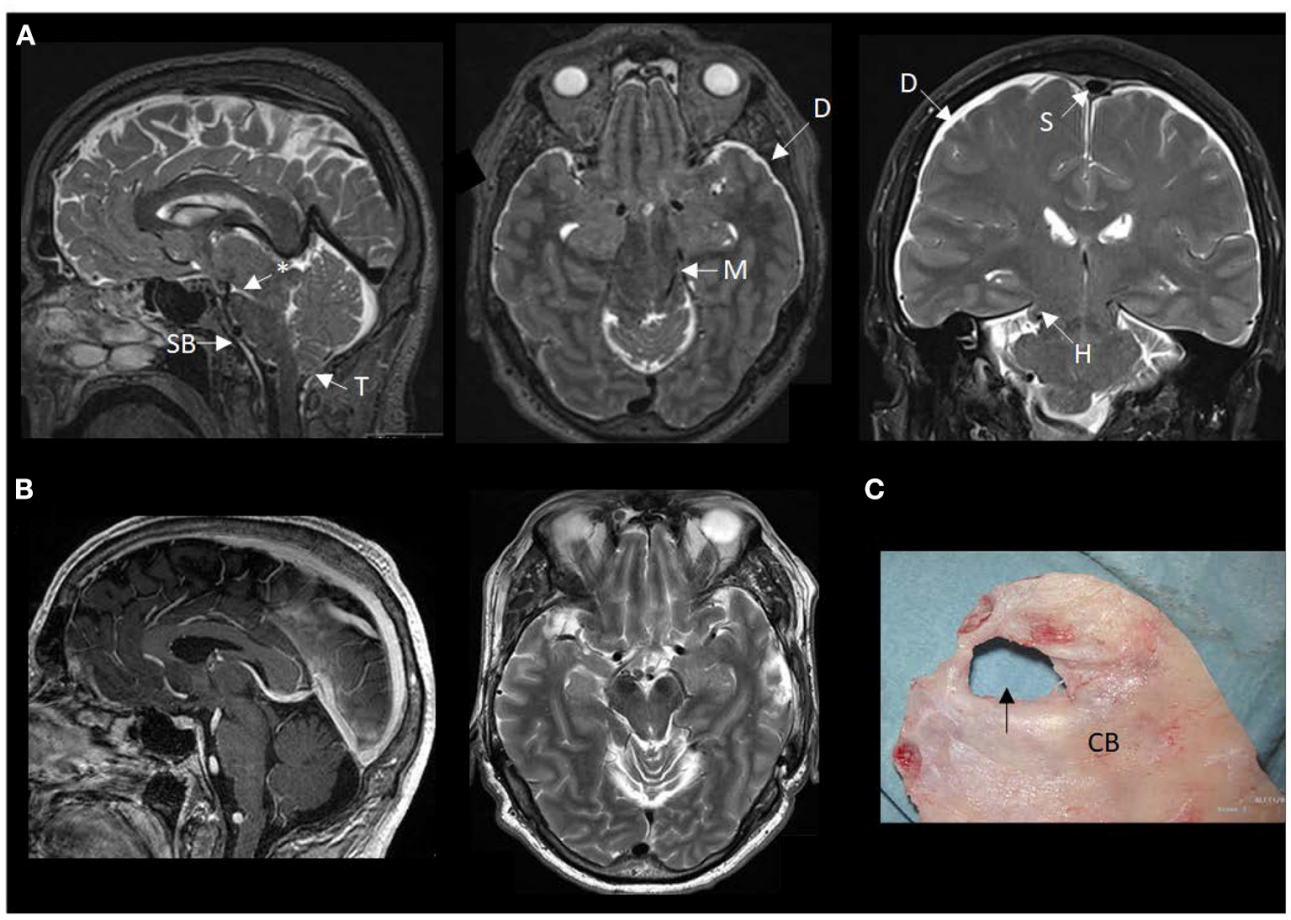
MRI findings of brain sagging dementia in this case. **(A)** The preoperative MRI in sagittal, axial and coronal planes show typical signs of intracranial hypotension, including sagging brainstem (SB) toward the clival bone, downward tonsillar (T) ectopy, smaller ponto-mesencephalic angle (indicated by an asterisk), enhancement of the dura (D), rounding of the cross-section of the dural venous sinus (S) indicative of dural venous engorgement, transtentorial herniation (H), and reduced space for the mesencephalon (M). **(B)** The postoperative MRI in sagittal and axial planes demonstrate reversal of the MRI signs of brain sagging. **(C)** The cranial bone (CB) with a defect (indicated by an arrow), as presented during surgical exploration.

**Figure 3:**
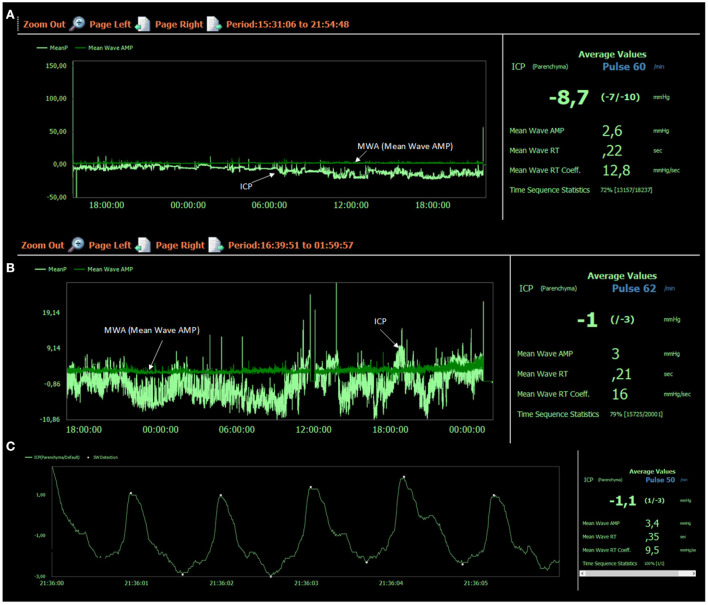
Intracranial pressure was measured from a Codman ICP sensor placed in the right frontal lobe. **(A)** The left window presents the trend plots of mean ICP (MeanP, light green) and mean ICP wave amplitude (MeanWave AMP, darker green). To the right is indicated the average values for the trend plots: Mean ICP – 8.7 mmHg, Mean Wave AMP (amplitude) 2.6 mmHg, Mean wave RT (Rise time) 0.22 s, Mean Wave RT Coeff (Rise time coefficient) 12.8 mmHg/s. Total 18,237 6-s windows were analyzed, wherein 13,157 were of acceptable quality for analysis (acceptance 72%). **(B)** The left window presents another trend plot of mean ICP (MeanP, light green) and of mean ICP wave amplitude (MeanWave AMP, darker green). To the right is indicated the average values for these trend plots: Mean ICP – 1.0 mmHg, Mean Wave AMP (amplitude) 3.0 mmHg, Mean wave RT (Rise time) 0.21 s, Mean Wave RT Coeff (Rise time coefficient) 16.0 mmHg/s. Total 20,001 6-s windows were analyzed, wherein 15,725 were of acceptable quality for analysis (acceptance 79%). **(C)** One 6-s time window is shown with five single pressure waves identified by their diastolic minimum and systolic maximum pressures. For this particular 6-s time window, parameters were: Mean ICP – 1.1 mmHg, Mean Wave AMP (amplitude) 3.4 mmHg, Mean wave RT (Rise time) 0.35 s, Mean Wave RT Coeff (Rise time coefficient) 9.5 mmHg/s. Image: Sensometrics RD Software (dPCom AS, Oslo, Norway).

During his stay in our department, imaging showed severe brain sagging with subsequent CSF flow obstruction through the cerebral aqueduct (Figure 2). A comprehensive search for CSF leakage was performed; dynamic computed tomography (CT) myelogram and repeated craniospinal MRI after administration of intrathecal gadobutrol (Gadovist, Bayer, GE), according to our department's protocol (5). However, no CSF leak was identified. Continuous intracranial pressure (ICP) monitoring revealed severe intracranial hypotension with ICP scores < − 10 mmHg (Figure 3). The mean wave amplitude (MWA) was within the normal range, suggesting no impaired intracranial compliance (6). The patient deteriorated further and developed severe antegrade amnesia, stereotyped and bizarre behavior, becoming socially and sexually inappropriate. At one point, he assaulted the nurse in his room, thus acquiring surveillance by a security guard. He lost insight, and his decision-making became seriously hampered. For instance, he insisted to divorce his wife. Mini-Mental exam score worsened in a matter of weeks from 26/30 to eventually a point where he could not cooperate for the test.

Due to his rapid clinical decline, a two-step cranial surgical approach was suggested, and his family consented. A cranial defect corresponding to his childhood head trauma was evident during the surgical exploration (Figure 2). The CSF leakage site was repaired, and an external ventricular drain was placed to overcome the supratentorial hydrocephalus induced by the brain sagging and subsequent CSF outflow obstruction. The splitting of the tentorium on the left side was also performed. The right tentorium was split in the second surgical step, and herniated brain tissue was resected. Despite a transient post-operative clinical improvement, his condition deteriorated. He became drowsy and showed clinical signs of herniation upon attempting to withdraw the drain. Therefore, he received a ventriculoperitoneal shunt with Codman-Hakim vale on a low setting (5 cmH_2_O).

His condition improved remarkably after shunt surgery, and he was discharged shortly thereafter. At 3-months follow-up, he was in good shape, with no neurological deficits and no more headaches. He scored 30/30 on MMSE and showed a good quality of life on the Short Form Health Survey (SF-36). He was back in his position as company director. He had no recollection of the time around his stay and was deeply sorry for his bizarre behavior. There was no sign of relapse on the 5-years follow-up.

## Discussion

The constellation of signs and symptoms of frontotemporal dementia caused by SIH, known as brain sagging dementia (BSD), has been increasingly recognized recently (7–9). The condition is twice as common in male patients and peaks in the sixth decade of life (3). Like in SIH, most patients present with some form of headache. In addition, BSD patients suffer from cognitive and behavioral changes that can potentially progress and severely impair their life.

Although a spinal CSF leakage is thought to be the etiology behind SIH and BSD, it is not radiologically evident in a significant number of patients (3, 4). In a recent review on BSD, a CSF leakage site was found in merely 13% of cases (3). Moreover, no cranial leak was reported in the two largest reviews on SIH and BSD (3, 4). Thus, this is the first report on a case of BSD caused by a cranial CSF leak.

This case report is remarkable for several reasons. First, it shows that a cranial cause of the leak must be explored thoroughly, particularly if no spinal leak is found. This is especially important as only the minority of leaks are successfully recognized in BSD patients (3). A thorough interview with the patient and their relatives may unveil details that can aid toward a potential CSF leakage site. In the current case, although no CSF leak was evident on radiological workup, the clinical picture, pathologically negative ICP, and past medical history with childhood cranial trauma with corresponding obscure radiological signs of old trauma in that area indicated surgical exploration.

Second, the complexity of this condition is well illustrated by the extended delay between the primary injury and fulminant disease with progressive signs and symptoms. Although the patient had some form of headache throughout the years, his condition severely escalated during the last 2 years, with complete decompensation within months. Moreover, the fact that CSF shunting was his ultimate remedy shows the coping mechanism his CSF circulatory system had to withstand until absolute decompensation.

Although the exact mechanism behind this can only be speculated at this moment, it is presumably multifactorial, involving both the compensatory mechanism and cranial to spinal fluid shift concept that has been proposed in patients with SIH (10, 11). In our patient, we believe that the decompensation was preceded by CSF depletion, causing the brain sagging, responsible for patients' initial BSD signs and symptoms, as described in great detail in a narrative review that we recently published (3). Further progress resulted in CSF outflow obstruction at the cranio-cervical junction, impeding the CSF flow system (12). This may have instigated further escalation of the now “enclosed intracranial compartment” with profound hypovolemia and hypotension evident on ICP monitoring, ultimately resulting in patients' critical deterioration. This theory is supported by the fact that the patient developed severe supratentorial hydrocephalus following the surgical repair of the cranial leak that required a CSF diversion procedure. Although the so-called “rebound intracranial hypertension” following treatment of SIH is well described in the literature (13, 14), in our patient with a CSF outflow obstruction at the cerebral aqueduct, it would be detrimental if left untreated.

Finally, this report highlights the importance of recognizing and treating this potentially reversible form of early-onset dementia, regardless of the condition's etiology, severity, and duration. Being familiar with the diagnostic criteria for BSD may be helpful in this process.

## Data availability statement

The raw data supporting the conclusions of this article will be made available by the authors, without undue reservation.

## Ethics statement

Ethical review and approval was not required for the study on human participants in accordance with the local legislation and institutional requirements. The patients/participants provided their written informed consent to participate in this study.

## Author contributions

Conceptualization and design, data analysis, review and editing, and approval of the final manuscript: AL and PE. Writing—original draft: AL. Supervision, administration, and material requests: PE. Both authors contributed to the article and approved the submitted version.

## Conflict of interest

The authors declare that the research was conducted in the absence of any commercial or financial relationships that could be construed as a potential conflict of interest.

## Publisher's note

All claims expressed in this article are solely those of the authors and do not necessarily represent those of their affiliated organizations, or those of the publisher, the editors and the reviewers. Any product that may be evaluated in this article, or claim that may be made by its manufacturer, is not guaranteed or endorsed by the publisher.
