# Supplementary material for: The first report on brain sagging dementia caused by a cranial leak: A case report
Source: Front Neurol. 2022 Sep 29;13:1006060. doi: 10.3389/fneur.2022.1006060 (PMC9556835; doi:10.3389/fneur.2022.1006060)
Supplement: Supplementary file 1 [file Data_Sheet_1.PDF]

Supplementary Material

The First Report on Brain Sagging Dementia Caused by A Cranial Leak: A Case Report

Aslan Lashkarivand[1,2], Per Kristian Eide[1,2*]

[1]Department of Neurosurgery, Oslo University Hospital – Rikshospitalet, Oslo, Norway
[2]Institute of Clinical Medicine, Faculty of Medicine, University of Oslo, Oslo, Norway

*Correspondence:
Professor Per Kristian Eide, MD PhD
Department of Neurosurgery
Oslo University Hospital - Rikshospitalet
Pb 4950 Nydalen,
N-0424 Oslo, Norway
p.k.eide@medisin.uio.no/peide@ous-hf.no

Content

eTable 1. Systematic Review Search Protocol………………………………………………...2
eFigure 1. Prisma 2009 Flow Diagram……………………………………………………..6

eTable 1. Systematic Review Search Protocol

Sources used for the search:

Database	03.05.2022
MEDLINE (Ovid):	509
Embase (Ovid):	1031
Cochrane Library:	21
PsycInfo (Ovid):	65
Number of references prior to duplicate deletion	1626
Number of references after duplicate deletion	1143

The search was initially performed on September 30th 2019, updated on April 21st 2021 and on 3rd of May 2022.
The search was conducted by Hilde Iren Flaatten, Medical Librarian, University of Oslo: Library of Medicine and Science. Literature searching and Evidence-Based Practice Unit

---

Database: Ovid MEDLINE(R) ALL

1 Intracranial Hypotension/ or (intracranial hypotension or intra-cranial hypotension or intracranial csf hypotension or intra-cranial csf hypotension or craniospinal hypotension).tw,kf.
2 exp Cerebrospinal Fluid Leak/ or ((CSF adj3 (leak* or hypovolemi* or hypovolaemi* or rhinorrhoea* or otorrhea*)) or (cerebr* adj3 (leak* or hypovolemi* or hypovolaemi* or rhinorrhoea*or otorrhea*)) or liquorr*).tw,kf.
3 ((low cerebro* adj2 pressure) or orthostatic headache* or postural headache*).tw,kf.
4 or/1-3
5 Dementia/ or cognition disorders/ or cognitive dysfunction/ or Neurocognitive Disorders/ or (dementia or cognit* or neurocognit* or behav* or (personal* adj2 change*)).tw,kf.
6 Apathy/ or Stupor/ or (apath* or disinhibition* or confusion* or disorientation* or desorientation* or stupor*).tw,kf.
7 memory disorders/ or exp amnesia/ or (memory or memories or amnesia).tw,kf.
8 communication disorders/ or language disorders/ or speech disorders/ or confusion/ or ((impair* or loss or disorder* or defect* or deficit* or dysfunction* or decline* or disturbance* or problem*) adj3 (language or speech or communication)).tw,kf.
9 psychology.fs. or (neuropsychological* or psychological* or mental state* or mental status).tw,kf.
10 or/5-9
11 4 and 10
12 ((brain adj3 sagging) or (brain adj3 sag) or (brain adj3 sagged) or (brain adj3 sink*) or (cerebr* adj3 sagging) or (cerebr* adj3 sag) or (cerebr* adj3 sagged)).tw,kf.
13 11 or 12
14 limit 13 to english language

Compressed search:

((Intracranial Hypotension/ or exp Cerebrospinal Fluid Leak/ or (intracranial hypotension or intra-cranial hypotension or intracranial csf hypotension or intra-cranial csf hypotension or craniospinal hypotension or (CSF adj3 (leak* or hypovolemi* or hypovolaemi* or rhinorrhoea* or otorrhea*)) or (cerebr* adj3 (leak* or hypovolemi* or hypovolaemi*)) or liquorrhea or (low cerebro* adj2 pressure) or orthostatic headache* or postural headache*).tw,kf.) and (Dementia/ or Cognition Disorders/ or Neurocognitive Disorders/ or Apathy/ or Stupor/ or memory disorders/ or exp amnesia/ or

communication disorders/ or language disorders/ or speech disorders/ or confusion/ or psychology.fs. or (dementia or cognit* or neurocognit* or behav* or (personal* adj2 change*) or apath* or disinhibition* or confusion* or disorientation* or desorientation* or stupor* or memory or memories or amnesia or neuropsychological* or psychological* or mental state* or mental status or ((impair* or loss or disorder* or defect* or deficit* or dysfunction* or decline* or disturbance* or problem*) adj3 (language or speech or communication))).tw,kf.)) or ((brain adj3 sagging) or (brain adj3 sag) or (brain adj3 sagged) or (brain adj3 sink*) or (cerebr* adj3 sagging) or (cerebr* adj3 sag) or (cerebr* adj3 sagged)).tw,kf.

Database: Embase Classic+Embase

1 intracranial hypotension/ or (intracranial hypotension or intra-cranial hypotension or intracranial csf hypotension or intra-cranial csf hypotension or craniospinal hypotension).tw,kf.
2 exp liquorrhea/ or ((CSF adj3 (leak* or hypovolemi* or hypovolaemi* or rhinorrhoea* or otorrhea*)) or (cerebr* adj3 (leak* or hypovolemi* or hypovolaemi* or rhinorrhoea* or otorrhea*)) or liquorr*).tw,kf.
3 ((low cerebro* adj2 pressure) or orthostatic headache* or postural headache*).tw,kf.
4 or/1-3
5 dementia/ or cognitive defect/ or mild cognitive impairment/ or exp confusion/ or "disorders of higher cerebral function"/ or disorientation/ or apathy/ or stupor/
6 behavior disorder/ or abnormal behavior/ or exp disruptive behavior/ or exp impulse control disorder/
7 (dementia or cognit* or neurocognit* or behav* or (personal* adj2 change*)).tw,kf.
8 (apath* or disinhibition* or confusion* or disorientation* or desorientation* or stupor*).tw,kw.
9 memory disorder/ or exp amnesia/ or (memory or memories or amnesia).tw,kf.
10 communication disorder/ or language disability/ or speech disorder/
11 ((impair* or loss or disorder* or defect* or deficit* or dysfunction* or decline* or disturbance* or problem*) adj3 (language or speech or communication)).tw,kf.
12 mental health/ or (neuropsychological* or psychological* or mental state* or mental status).tw,kf.
13 or/5-12
14 4 and 13
15 ((brain adj3 sagging) or (brain adj3 sag) or (brain adj3 sagged) or (brain adj3 sink*) or (cerebr* adj3 sagging) or (cerebr* adj3 sag) or (cerebr* adj3 sagged)).tw,kf.
16 14 or 15
17 limit 16 to conference abstract
18 16 not 17
19 limit 18 to english language

Database: Cochrane Library
https://bit.ly/2n5DWth

#1 MeSH descriptor: [Intracranial Hypotension] this term only
#2 (intracranial NEXT hypotension or intra NEXT cranial NEXT hypotension or intracranial NEXT csf NEXT hypotension or intra NEXT cranial NEXT csf NEXT hypotension):ti,ab,kw OR (or craniospinal NEXT hypotension):ti,ab,kw (Word variations have been searched)
#3 MeSH descriptor: [Cerebrospinal Fluid Leak] explode all trees

#4 (CSF NEAR/2 (leak* or hypovolemi* or hypovolaemi* or rhinorrhoea* or otorrhea*)):ti,ab,kw OR (cerebr* NEAR/2 (leak* or hypovolemi* or hypovolaemi* or rhinorrhoea* or otorrhea*)):ti,ab,kw OR (liquorr*):ti,ab,kw (Word variations have been searched)
#5 (low cerebro* NEAR/1 pressure):ti,ab,kw OR (orthostatic NEXT headache*):ti,ab,kw OR (postural NEXT headache*):ti,ab,kw (Word variations have been searched)
#6 {OR #1-#5}
#7 MeSH descriptor: [Dementia] this term only
#8 MeSH descriptor: [Cognition Disorders] this term only
#9 MeSH descriptor: [Cognitive Dysfunction] this term only
#10 MeSH descriptor: [Neurocognitive Disorders] this term only
#11 (dementia or cognit* or neurocognit* or behav*):ti,ab,kw OR (personal* NEAR/1 change*):ti,ab,kw (Word variations have been searched)
#12 MeSH descriptor: [Apathy] this term only
#13 MeSH descriptor: [Stupor] this term only
#14 (apath* or disinhibition* or confusion* or disorientation* or desorientation* or stupor*):ti,ab,kw (Word variations have been searched)
#15 MeSH descriptor: [Memory Disorders] this term only
#16 MeSH descriptor: [Amnesia] explode all trees
#17 (memory or memories or amnesia):ti,ab,kw (Word variations have been searched)
#18 MeSH descriptor: [Communication Disorders] this term only
#19 MeSH descriptor: [Language Disorders] this term only
#20 MeSH descriptor: [Speech Disorders] this term only
#21 MeSH descriptor: [Confusion] this term only
#22 ((impair* or loss or disorder* or defect* or deficit* or dysfunction* or decline* or disturbance* or problem*) NEAR/2 (language or speech or communication)):ti,ab,kw (Word variations have been searched)
#23 MeSH descriptor: [] explode all trees and with qualifier(s): [psychology - PX]
#24 (neuropsychological* or psychological* or (mental NEXT state*) or (mental NEXT status)):ti,ab,kw (Word variations have been searched)
#25 {OR #7-#24}
#26 #6 AND #25
#27 ((brain NEAR/2 sagging) or (brain NEAR/2 sag) or (brain NEAR/2 sagged) or (brain NEAR/2 sink*) or (cerebr* NEAR/2 sagging) or (cerebr* NEAR/2 sag) or (cerebr* NEAR/2 sagged)):ti,ab,kw (Word variations have been searched)
#28 #26 OR #27

Database: PsycINFO

1 (intracranial hypotension or intra-cranial hypotension or intracranial csf hypotension or intra-cranial csf hypotension or craniospinal hypotension).mp.
2 ((CSF adj3 (leak* or hypovolemi* or hypovolaemi* or rhinorrhoea* or otorrhea*)) or (cerebr* adj3 (leak* or hypovolemi* or hypovolaemi* or rhinorrhoea* or otorrhea*)) or liquorr*).mp.
3 ((low cerebro* adj2 pressure) or orthostatic headache* or postural headache*).mp.
4 or/1-3
5 (dementia or cognit* or neurocognit* or behav* or (personal* adj2 change*)).mp.
6 (apath* or disinhibition* or confusion* or disorientation* or desorientation* or stupor*).mp.
7 (neuropsychological* or psychological* or mental state* or mental status).mp.
8 (memory or memories or amnesia).mp.
9 ((impair* or loss or disorder* or defect* or deficit* or dysfunction* or decline* or disturbance* or problem*) adj3 (language or speech or communication)).mp.

10 or/5-9
11 4 and 10
12 ((brain adj3 sagging) or (brain adj3 sag) or (brain adj3 sagged) or (brain adj3 sink*) or (cerebr* adj3 sagging) or (cerebr* adj3 sag) or (cerebr* adj3 sagged)).mp.
13 11 or 12
14 limit 13 to english language

----

eFigure 1. PRISMA 2009 Flow Diagram

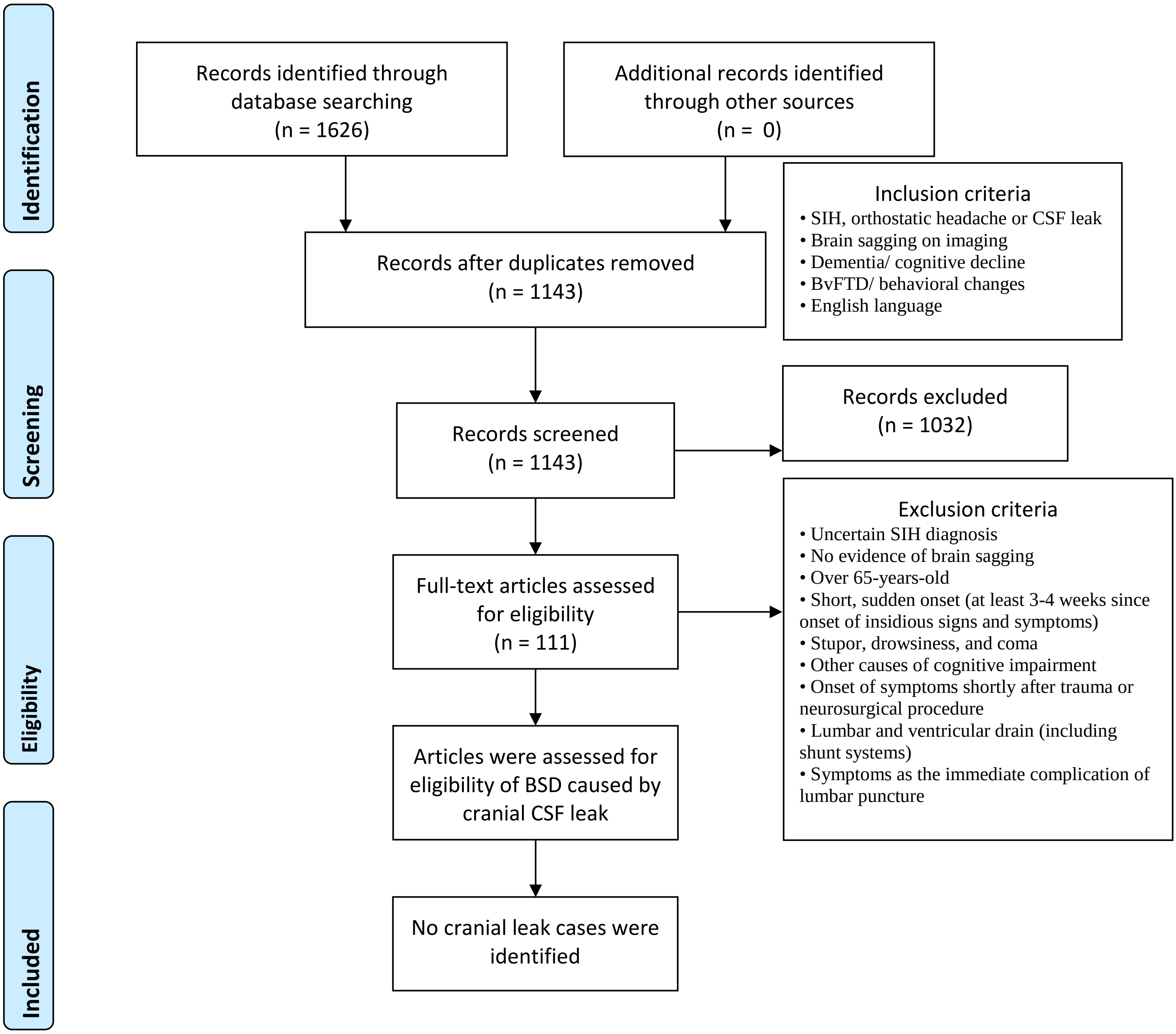

From: Moher D, Liberati A, Tetzlaff J, Altman DG, The PRISMA Group (2009). *P*referred *R*eporting *I*tems for *S*ystematic Reviews and *M*eta-*A*nalyses: The PRISMA Statement. PLoS Med 6(7): e1000097. doi:10.1371/journal.pmed1000097

For more information, visit www.prisma-statement.org.
